# Age differences in the association between stressful work and sickness absence among full-time employed workers: evidence from the German socio-economic panel

**DOI:** 10.1007/s00420-018-1298-3

**Published:** 2018-02-28

**Authors:** Simon Götz, Hanno Hoven, Andreas Müller, Nico Dragano, Morten Wahrendorf

**Affiliations:** 1 Institute of Medical Sociology, Centre for Health and Society, Medical Faculty, University of Duesseldorf, Universitaetsstrasse 1, 40225 Duesseldorf, Germany; 2 Institute of Psychology, Work and Organizational Psychology, University of Duisburg-Essen, Universitätsstrasse 2, 45141 Essen, Germany

**Keywords:** Work stress, Effort–reward imbalance, Sickness absence, Age differences, GSOEP

## Abstract

**Purpose:**

We aim to extend current knowledge on associations between stressful work and sickness absence, first, by studying associations between ERI and sickness absence among full-time employees from various occupations, and second, by investigating if associations vary by age.

**Methods:**

We use data from four waves of the German socio-economic panel (GSOEP), collected among men and women between 2006 and 2012, with 9418 observations. Stressful work is measured with a short form of the ERI questionnaire. We investigate an imbalance between effort and reward (ER ratio) as well as the two main components (“high effort” and “low reward”). Sickness absence is measured by self-reported number of sickness days (assessed the following year). After descriptive analyses, we estimate a series of multivariable regressions, including tests for interactions between age and work stress.

**Results:**

Each of the three indicators of stressful work is related to higher number of sickness days, with except of “high effort” in case of men. Findings remain significant after adjusting for social position (income, education and occupational class) and health. In addition, for both men and women, associations were slightly higher among older workers, though interactions did not reach statistical significance.

**Conclusion:**

Our findings support that stressful work is linked to sickness absence across a wide spectrum of jobs with varying incomes and educational levels, and also that associations are slightly more pronounced among older workers.

**Electronic supplementary material:**

The online version of this article (10.1007/s00420-018-1298-3) contains supplementary material, which is available to authorized users.

## Introduction

Sickness-related absence from work is a major concern of today’s labour markets and ageing workforces in Europe. For example, according to Eurofound, the average rate of sickness absence in the European Union varies between 3 and 6% (Eurofound 2010). This has considerable consequences for companies and national health policies, as it decreases productivity and increases costs for health insurances. Studying predictors of sickness absence, therefore, is important, as it helps to identify factors related to sickness absence and to develop workplace health interventions.

Studies from several countries have identified different factors that are related to sickness-related absence from work (Beemsterboer et al. 2009; Harrison and Martocchio 1998), including sociodemographic factors and psychosocial working conditions. When it comes to sociodemographic factors, for example, findings show that older workers have generally more days of sickness absence than younger workers (Donders et al. 2012; Taimela et al. 2007). Studies also highlight that patterns and reasons for sickness absence differ between women and men, with levels being generally higher among women—a finding that may be related to differing significance of the work role (Casini et al. 2013; Krantz and Lundberg 2006; Messing et al. 2003; Siegrist et al. 2006; Sterud 2014). Stressful working conditions are another factor related to sickness absence, mainly because of their health-related consequences. This was shown by various occupational cohort studies. Thereby, measures of stressful work range from established theoretical models, such as job strain (Ala-Mursula et al. 2005; Mortensen et al. 2017), effort–reward imbalance (Ala-Mursula et al. 2005; Derycke et al. 2013; du Prel et al. 2015; Fahlén et al. 2009; Lidwall 2016; Schreuder et al. 2010) or relational injustice (Head et al. 2007), to single stressors at work (Brussig and Ahlers 2007). Yet, despite this consistent evidence linking work stress to sickness absence, studies are generally based on rather homogenous occupational cohorts, such as nurses (Farquharson et al. 2012; Schreuder et al. 2010), transport operators (Cunradi et al. 2005), teachers (Derycke et al. 2013), university employees (Donders et al. 2012) or civil servants (Head et al. 2007). Similarly, most cohorts are recruited during midlife, and thus, older workers are clearly underrepresented in existing studies. This focus on midlife and on homogenous occupational groups, however, has at least two consequences for scientific knowledge about the association between stressful work and sickness absence.

First, more studies are needed that establish the links between work stress and sickness absence across various occupational groups that cover a wide spectrum of jobs with varying incomes and educational levels. This would help to rule out that associations between stressful work and sickness absence are due to cohort specific characteristics. For example, options and regulations for sickness absence may differ between various occupations, and thus, the links between work stress and sickness absence may vary as well. Therefore, the first aim of the present study is to investigate links between stressful work and sickness absence among the full-time employees from various occupations based on a general population survey.

Second, because of demographic changes and ageing workforces in Europe, it is necessary to extend studies to older workers. This, notably, not just concerns the general question if the overall level of sickness absence differs by age, but also the more specific question if the association between stressful work and sickness absence varies by age. In fact, there is evidence that the impact of stressful work differs depending on the period, or life stage, at which it occurs (Burr et al. 2017; Donders et al. 2012; Payne and Doyal 2010; Sampaio and Augusto 2012; Shultz 2010). Notably, this is in line with an important principle of life course research, which is to consider the timing of an exposure to understand its health-related consequences in more details (Ben-Shlomo and Kuh 2002; Wahrendorf and Chandola 2016). Older persons, for example, may be more vulnerable to work stress, because the ageing process is accompanied by changing coping capabilities and resources (Hobfoll 1989; Lazarus and DeLongis 1983), as well as changes of the physiological system. Older people, therefore, may be more likely to turn sick in case of stressful work, as well as they may take longer to recover. In that case, the association between stress and sickness absence would be more pronounced for older workers. A stronger association for older workers though could also have indirect reasons, because younger workers have possibly different motivations to work than older workers. For example, younger workers may face higher pressure to develop strong ties to the labour market, and therefore, they are more likely to continue working compared to their older counterparts, even if conditions at work are poor. There are, however, also reasons why associations between stress at work and sickness absence could be less pronounced for older workers than for younger workers. For example, older workers may face more difficulties to find a new job in case of job loss, and therefore, they are probably more likely to tolerate adverse conditions than younger workers.

Overall, there are numerous, and partly divergent assumptions on how associations between work stress and sickness absence may differ by age, but evidence on this is lacking. The second aim of this study, thus, is to compare links between work stress and sickness absence between different age groups. In sum, this leads to the following research questions:

1. Is stressful work associated to sickness-related absence from work for men and women?
2. If so, does the association between stressful work and sickness-related absence from work differ by age for men and women?

## Methods

### Data

We base our study on the German socio-economic panel (GSOEP) (Schupp et al. 2016). GSOEP is the largest panel study in Germany, based on a random sample of private households. It started 1984 in western Germany with 12,245 respondents from 5921 households with on-going waves of data collection ever since. Data is collected for each household member aged 18 or older, using paper and pencil interviews (PAPI), computer assisted personal interviews (CAPI) and self-completion questionnaires. The response rate in wave 1 was 62%, and the attrition rate between wave 1 and wave 2 was 14% (for more information see: Kroh et al. 2017). In 1990, eastern Germany joined the study. Furthermore, additional subsamples were added in the course of the study to maintain the population representation and to increase the sample size (especially in 2000). At present, GSOEP provides representative data for more than 20,000 adult men and women in Germany taken from nearly 11,000 households. Besides sociodemographic characteristics, this includes information on individual living conditions, work and employment, income, health and sickness-related absence from work. (For more information on the GSOEP data see: Wagner et al. 2007). One of the main advantages of the GSOEP is the longitudinal nature of the information, where information on work stress (available in 2006 and 2011) can be linked to number of sickness days, as collected the year after (2007 and 2012, respectively). For the present study, we focused on men and women aged 18–65 years in the year of work stress assessment (33,648 observations) and applied the following sample restrictions: First, we excluded those who were not working and had no information on sickness absence for the year after (12,994 observations). Second, we excluded those who were working in part-time (5719 observations), because part-time workers often not work five days the week, making comparisons of days in sickness absence with full-time workers impossible. Third, we excluded people who either were in vocational training or in military service (850 observations), because employment relations and salary regulations are different for these groups. Fourth, people who changed their job during the observation period were also excluded (981 observations), because the association between work stress and sickness absence days is unclear. Fifth, we excluded people who were self-employed (1677 observations), as self-employed workers are not automatically qualified for statutory sick pay in case of sickness. Sixth, we decided to exclude people who were permanently sick (with more than 200 days of sickness absence, 57 observations), since they hardly participated on the labour market. Finally, among these remaining 11,370 observations, we restricted the sample to those with complete information on all variables under study (excluding another 1952 observations), with no indication of systematic missings. In sum, this results in a final sample of 9418 observations (person-year observations) with complete data on all study variables (based on 7193 individuals, each observed on average in 1.3 observation periods).

### Measures

#### Work stress

Work stress is measured by the short version of the ERI questionnaire, as validated by a previous study for the GSOEP (Siegrist et al. 2009). The ERI model identifies stressful work in terms of an imbalance between high efforts spent at work and low rewards received in turn (Siegrist and Wahrendorf 2016). The short version of the questionnaire includes three items for effort and seven items for reward. Items for effort refer to perceived psychological demands at work, and reward includes salary, esteem, job security and career opportunities. Each item is listed in Supplementary Table S1. In GSOEP, items are rated on a five-point scale, ranging from ‘disagree’, via ‘agree, and I am somewhat distressed’, to ‘agree, and I am very distressed’. For the analyses, we followed established procedures and created sum-scores for effort and reward, as well as we calculate the ratio between effort and reward (adjusted for number of items). On this basis, we first create two binary indicators for each of the two main components. Specifically, to identify elevated levels of work stress, people who belonged to the highest tertile of the effort-scale were classified as “high effort” and those in the lowest tertile of the reward scale as “low reward” (in both cases tertiles are based on the total sample in work). Then, effort–reward imbalance was calculated by dividing the sum score of the ‘effort’ items (nominator) through the sum score of the ‘reward’ items (adjusted for number of items; denominator). This results in a sum score where higher values are related to higher levels of work stress. For the analyses, an imbalance was assumed in case values are higher than 1 (labelled as “ER ratio > 1”). In sum, this leads to three different binary indicators of stressful work. More details on psychometric properties for GSOEP (Siegrist et al. 2009) and on the conceptual basis are fully described elsewhere (Siegrist 2016).

#### Sickness days

To measure sickness-related absence from work, the present study relies on the total number of days of absence from work. More specifically, in the year following the assessment of work stress respondents answered an open question on how many days they were not able to work because of illness in the previous year. In contrast to other approaches focusing on number of absence episodes, this reflects the total absence duration in 1 year (possibly based on several episodes) (Steel 2003).

#### Age groups

We distinguish four age groups for the analyses, each covering a distinct phase in the life course (Willis and Martin 2005). The first group, “Job starters” (Age 18 till a 29), covers the period where people make first experiences on the labour market. Next, “early midlife” (age 30–45) and “late midlife” (age 46–57) refer to the main phase of working life, accompanied by increasing responsibilities at work and parenthood, and progressing ageing processes. Lastly, “older working life” (58–65 years) represents those who approach the end of working life.

#### Additional measures

We also include two sociodemographic measures (partnership and number of young children), three indicators of the respondent’s social position (education, income and occupational position), and self-rated health. In the case of partnership, we use a binary indicator of whether the respondents live with a partner (regardless of the marital status). The number of young children (aged 14 or younger) is regrouped into “none”, “1” and “2 or more”. As an indicator of education, we use the total years spent in full-time education. Income is based on the monthly household income that we adjusted for household size in accordance with the OECD equivalent-scale (Burniaux et al. 1998), and thereafter regrouped into income tertiles (“high”, “medium” and “low”). Occupational position is measured according to the Erikson–Goldthorpe–Portocarero scheme (EGP scheme) (Erikson and Goldthorpe 1992). This scheme classifies occupations into seven classes based on specific aspects under which a person performs work on the labour market, or more specifically, “employment relations”. For the analyses, occupations were regrouped into four categories: “upper service class” (EGP I), “lower service class” (EGP II), “routine non-manuals workers” (EGP III, IVab), and “skilled and unskilled manual workers” (EGP IVc, V, VI, VII). Self-rated health was measured by a single question (“How would you describe your current health?”) with five categories ranging from “very good” to “bad”. Answers were dichotomized into “good or better” and “less than good”. An overview of all measures is presented in Table 1.

**Table 1:** Sample description: observations (No.) and percentage (%) or mean and standard deviation (SD): *n* = 9418

	Range or categories	No. or (mean)	% or (SD)
Sex	Male	6257	66.4
	Female	3161	33.6
Age groups	Job starters (18–29)	1036	11.0
	Early midlife (30–45)	3894	41.3
	Late midlife (46–57)	3511	37.3
	Older working life (58–65)	977	10.4
Sickness days	Range: 0–200 days	(9.1)	(20.0)
High effort	Yes	2714	28.8
	No	6704	71.2
Low reward	Yes	3639	38.6
	No	5779	61.4
ER ratio > 1	Yes	1391	14.8
	No	8027	85.2
Occupational position	Higher service class	1739	18.5
	Lower service class	2721	28.9
	Routine non-manuals	1682	17.9
	Skilled and unskilled manual workers	3276	34.8
Years in job	Range: 0–50 years	(13.6)	(10.5)
Income	High	4606	48.9
	Medium	3369	35.8
	Low	1443	15.3
Education years	Range: 7–18 years	(12.9)	(2.8)
Number of children < 14	None	6923	73.5
	1	1347	14.3
	2 or more	1148	12.2
Partnership	Living with partner	7937	84.3
	Living as single	1481	15.7
Self-rated health	Good or better	5457	57.9
	Less than good	3961	42.1
Total		9418	100.0

### Analytical strategy

We start with a basic sample description (Table 1). Thereafter, Table 2 explores how the three measures of work stress and number of sickness days are distributed by covariates. We also report tests of significance based on Chi-square, Wilcoxon–Mann–Whitney, or Kruskal–Wallis tests. Then, a first picture of the associations between work stress and sickness absence, and their variations by age, is presented in Table 3. Specifically, we show the mean number and the median of sickness days for each age group separately, including confidence intervals (95%), interquartile ranges (IQR) and tests of significance (Wilcoxon–Mann–Whitney test). In this case (and in later multivariable regressions), analyses consider sex differences and are conducted for men and women separately (Casini et al. 2013; Messing et al. 2003).

**Table 2:** High levels of stress at work (in percent) and sickness days [mean values, standard deviation, median and interquartile range (IQR)] by covariates: *n* = 9418

	High efforts	Low reward	ER ratio > 1	Sickness days			
	%	%	%	Mean (SD)		Median	IQR
Sex							
Male	28.1	37.0	13.8	8.6	(19.6)	2	10
Female	30.3	41.8	16.7	10.3	(20.8)	4	10
Age groups							
Job starters (18–29)	22.4	30.2	9.7	7.3	(15.3)	4	8
Early midlife (30–45)	29.0	40.8	15.5	8.0	(18.1)	3	10
Late midlife (46–57)	31.4	40.9	16.3	10.0	(21.2)	3	10
Older working life (58–65)	25.4	30.8	11.5	12.5	(25.9)	3	14
Occupational position							
Higher service class	37.8	30.9	15.1	6.6	(15.5)	2	6
Lower service class	30.8	37.5	15.1	8.7	(18.6)	3	10
Routine non-manuals	30.1	40.6	16.0	9.0	(20.0)	3	10
Skilled and unskilled manual workers	21.7	42.6	13.7	10.9	(22.9)	3	11
Income							
High	32.8	34.0	14.0	8.4	(18.7)	3	10
Medium	26.8	42.7	15.7	9.8	(20.8)	3	10
Low	20.9	43.9	15.1	10.0	(22.0)	3	10
Number of children < 14							
None	29.1	38.5	14.8	9.7	(21.1)	3	10
1	29.0	41.4	16.2	8.9	(19.1)	3	10
2 or more	26.8	36.3	12.6	6.5	(12.9)	3	8
Partnership							
Living with partner	29.2	38.7	14.7	9.1	(20.4)	3	10
Living as single	26.9	38.4	15.3	9.2	(19.9)	3	10
Self-rated health							
Good or better	23.4	31.0	9.7	5.8	(13.4)	2	6
Less than good	36.2	49.2	21.8	13.7	(25.8)	5	14
Total	28.8	38.6	14.8	9.1	(20.0)	3	10

**Table 3:** Sickness days by stress at work: divided by age groups for men and women: mean, standard deviation (SD), 95% CI, median (Med) and *p* values (based on Wilcoxon–Mann–Whitney test)

	Job starters (18–29)					Early midlife (30–45)					Late midlife (46–57)					Older working life (58–65)					All age Groups				
	Mean	(SD)	[95% CI]	Med	*p*	Mean	(SD)	[95% CI]	Med	*p*	Mean	(SD)	[95% CI]	Med	*p*	Mean	(SD)	[95% CI]	Med	*p*	Mean	(SD)	[95% CI]	Med	*p*
Women																									
High effort					0.670					0.022					< 0.001					0.072					< 0.001
Yes	10.4	(24.7)	[5.9–14.9]	5		12.3	(25.1)	[9.7–14.8]	5		14.1	(25.8)	[11.5–16.7]	5		18.7	(33.1)	[11.4–26.0]	7		13.3	(26.1)	[11.6–15.0]	5	
No	7.7	(13.5)	[6.3–9.1]	4		8.5	(17.0)	[7.4–9.7]	4		9.8	(19.9)	[8.4–11.2]	4		10.3	(19.6)	[7.5–13.1]	3		9.0	(17.8)	[8.2–9.8]	4	
Low reward					0.012					< 0.001					< 0.001					0.216					< 0.001
Yes	10.4	(21.1)	[7.2–13.6]	5		12.4	(24.5)	[10.3–14.5]	5		14.6	(26.9)	[12.3–16.9]	5		16.7	(30.6)	[10.2–23.2]	5		13.3	(25.6)	[11.9–14.7]	5	
No	7.2	(13.8)	[5.7–8.7]	4		7.6	(15.1)	[6.4–8.7]	3		8.5	(16.8)	[7.1–9.8]	3		10.9	(21.1)	[7.8–14.1]	4		8.1	(16.2)	[7.4–8.9]	3	
ER ratio > 1					0.785					0.003					< 0.001					0.017					< 0.001
Yes	11.0	(25.3)	[4.6–17.4]	5		14.0	(25.8)	[10.4–17.5]	5		17.0	(29.7)	[13.1–20.9]	5		24.4	(38.7)	[12.3–36.4]	10		15.7	(28.7)	[13.2–18.2]	5	
No	8.0	(15.3)	[6.5–9.4]	5		8.8	(18.3)	[7.6–9.9]	4		9.9	(19.7)	[8.6–11.1]	4		10.8	(20.8)	[8.1–13.5]	3		9.2	(18.6)	[8.5–10.0]	4	
Men																									
High effort					0.106					0.701					0.129					0.333					0.510
Yes	5.5	(13.7)	[3.0–8.0]	2		7.6	(18.8)	[6.3–9.0]	2		9.5	(18.6)	[8.1–10.8]	3		14.1	(29.6)	[9.6–18.7]	4		8.8	(19.8)	[7.9–9.8]	2	
No	6.6	(13.7)	[5.3–7.9]	3		7.1	(16.5)	[6.4–7.9]	3		9.4	(21.6)	[8.3–10.5]	2		11.8	(25.3)	[9.7–13.9]	2		8.5	(19.5)	[7.9–9.0]	2	
Low reward					0.122					< 0.001					< 0.001					0.015					< 0.001
Yes	6.1	(8.3)	[4.7–7.4]	4		9.5	(20.3)	[8.3–10.7]	4		11.0	(20.8)	[9.7–12.4]	4		15.2	(28.2)	[11.4–19.1]	5		10.4	(20.9)	[9.5–11.3]	4	
No	6.5	(15.2)	[5.0–8.0]	2		5.8	(14.6)	[5.1–6.6]	2		8.4	(20.6)	[7.3–9.4]	2		11.1	(25.5)	[8.9–13.4]	2		7.5	(18.6)	[6.9–8.1]	2	
ER ratio > 1					0.816					< 0.001					0.001					0.006					< 0.001
Yes	5.0	(8.5)	[2.5–7.4]	3		11.1	(24.4)	[8.8–13.5]	5		11.5	(21.4)	[9.3–13.7]	4		18.6	(34.0)	[10.6–26.5]	5		11.6	(23.7)	[10.0–13.2]	5	
No	6.5	(14.1)	[5.3–7.7]	3		6.6	(15.5)	[6.0–7.3]	2		9.0	(20.6)	[8.1–10.0]	2		11.7	(25.3)	[9.7–13.6]	2		8.1	(18.8)	[7.6–8.6]	2	

Next, we estimate a series of multivariable regression models using sickness days as dependent variable—again for each age group separately, as well as for all ages (Table 4). Importantly, these models consider the hierarchical structure of our data, that is, that some observations (level 1) are not independent, as they come from the same respondent nested in different survey years (level 2). In these models, the constant is allowed to vary within individuals (also called random intercept model for longitudinal data or “random-effect model” for panel data) (Andreß et al. 2013; Giesselmann and Windzio 2012). Unlike a “fixed-effect model” for panel data, this allows to include time constant predictors (e.g. education, income and occupational position). In addition, models are both presented for non-transformed (for ease of interpretation) and transformed sickness days, because sickness days were not normally distributed. For this, we compared different transformations and decided to adopt a square root transformation (Stoto and Emerson 1983). In sum, we present estimates of three regression models, estimated for each measure of work stress separately (maximum likelihood estimation). Model 1 presents estimates that are adjusted for partnership situation, years in job and number of young children. After checking for potential multicollinearity, model 2 adds education, income and occupational position, and thus, looks if associations remain consistent after accounting for social position. Model 3 then includes health to investigate a possible mediation via health (or confounding). Based on these regression models, we can already compare the effect sizes between the age groups. Then, to formally test interactions between work stress and age groups, we use the total sample and include interaction terms between work stress and each age group into the model. We hereby rely on model 2 (adjusting for sociodemographic factors and social position) and do not include health as potential mediator (or intermediate variable on the causal path between work stress and sickness absence) to avoid overadjustment. By comparing models without and with interactions on the basis of a likelihood-ratio test, we test for significant interactions (Mitchell 2012).

**Table 4:** Association between stress at work and sickness days

	Job starters (18–29)			Early midlife (30–45)			Late midlife (46–57)			Older working life (58–65)			All age groups		
	*b*	[95% CI]	*p*	*b*	[95% CI]	*p*	*b*	[95% CI]	*p*	*b*	[95% CI]	*p*	*b*	[95% CI]	*p*
Women															
High effort															
Model 1	2.95	[− 0.49–6.40]	0.093	3.87	[1.52–6.23]	0.001	4.38	[1.70–7.05]	0.001	8.47	[2.12–14.80]	0.009	4.19	[2.61–5.77]	< 0.001
Model 2	2.96	[− 0.50–6.42]	0.093	4.31	[1.95–6.66]	< 0.001	4.96	[2.29–7.64]	< 0.001	8.89	[2.48–15.30]	0.007	4.65	[3.07–6.24]	< 0.001
Model 3	2.55	[− 0.59–5.69]	0.111	3.33	[0.97–5.69]	0.006	3.26	[0.58–5.94]	0.017	7.43	[1.26–13.60]	0.018	3.32	[1.74–4.90]	< 0.001
Low reward															
Model 1	3.28	[0.16–6.39]	0.039	4.24	[2.04–6.43]	< 0.001	6.10	[3.59–8.62]	< 0.001	5.73	[− 0.58–12.10]	0.075	5.04	[3.57–6.51]	< 0.001
Model 2	3.22	[0.21–6.23]	0.036	4.06	[1.87–6.25]	< 0.001	5.80	[3.29–8.31]	< 0.001	6.14	[− 0.19–12.50]	0.057	4.85	[3.38–6.32]	< 0.001
Model 3	2.52	[− 0.48–5.52]	0.099	3.19	[1.01–5.37]	0.004	4.39	[1.88–6.90]	0.001	3.34	[− 2.82–9.51]	0.288	3.56	[2.09–5.02]	< 0.001
ER ratio > 1															
Model 1	3.54	[− 1.07–8.15]	0.133	5.16	[2.24–8.09]	0.001	7.18	[3.95–10.4]	< 0.001	13.4	[5.35–21.40]	0.001	6.26	[4.32–8.20]	< 0.001
Model 2	3.22	[− 1.36–7.79]	0.169	5.30	[2.39–8.22]	< 0.001	7.35	[4.13–10.6]	< 0.001	13.7	[5.68–21.70]	0.001	6.40	[4.46–8.33]	< 0.001
Model 3	3.74	[− 0.91–8.39]	0.115	3.70	[0.76–6.63]	0.014	5.43	[2.20–8.66]	0.001	10.3	[2.40–18.10]	0.011	4.56	[2.63–6.50]	< 0.001
Men															
High effort															
Model 1	− 1.27	[− 4.21–1.66]	0.394	0.46	[− 1.00–1.91]	0.536	− 0.08	[− 1.91–1.75]	0.931	2.47	[− 2.11–7.05]	0.291	0.28	[− 0.80–1.37]	0.608
Model 2	− 1.16	[− 4.02–1.70]	0.427	1.01	[− 0.45–2.46]	0.175	0.41	[− 1.43–2.24]	0.664	3.84	[− 0.73–8.41]	0.099	0.86	[− 0.23–1.95]	0.123
Model 3	− 1.47	[− 4.34–1.40]	0.315	0.33	[− 1.12–1.78]	0.654	− 0.76	[− 2.59–1.07]	0.415	2.44	[− 2.07–6.96]	0.289	− 0.059	[− 1.15–1.03]	0.916
Low reward															
Model 1	− 0.19	[− 2.97–2.58]	0.891	3.53	[2.19–4.86]	< 0.001	2.50	[0.77–4.23]	0.005	3.67	[− 0.60–7.95]	0.092	2.88	[1.87–3.90]	< 0.001
Model 2	− 0.60	[− 3.24–2.05]	0.657	3.03	[1.69–4.37]	< 0.001	2.14	[0.41–3.88]	0.015	2.85	[− 1.40–7.10]	0.188	2.46	[1.44–3.48]	< 0.001
Model 3	− 1.12	[− 3.81–1.57]	0.414	2.17	[0.82–3.53]	0.002	1.11	[− 0.62–2.84]	0.210	1.07	[− 3.15–5.29]	0.620	1.44	[0.42–2.45]	0.006
ER ratio > 1															
Model 1	− 1.66	[− 6.08–2.77]	0.463	4.43	[2.59–6.27]	< 0.001	2.34	[0.00–4.68]	0.050	6.20	[− 0.29–12.7]	0.061	3.33	[1.92–4.75]	< 0.001
Model 2	− 1.81	[− 6.17–2.56]	0.417	4.19	[2.36–6.03]	< 0.001	2.38	[0.05–4.71]	0.045	6.51	[0.09–12.90]	0.047	3.33	[1.92–4.74]	< 0.001
Model 3	− 2.66	[− 7.06–1.75]	0.237	3.14	[1.29–4.98]	0.001	1.13	[− 1.19–3.45]	0.340	4.70	[− 1.64–11.00]	0.146	2.09	[0.68–3.49]	0.004

Results of multivariable linear regression analyses predicting number of sickness days: regression coefficients (*b*), 95% confidence intervals [95% CI] and *p* values (*p*)

The presented estimates (*b*) correspond to the adjusted mean differences between those with and without stress. All estimations are based on random effect models accounting for year of data collection. Model 1 adjusts for partnership, years in current job and number of children living in the household (and age in case of all age groups). Model 2 is additionally adjusted for occupational position, education and income. Model 3 additionally includes self-rated health

**Fig. 1:**
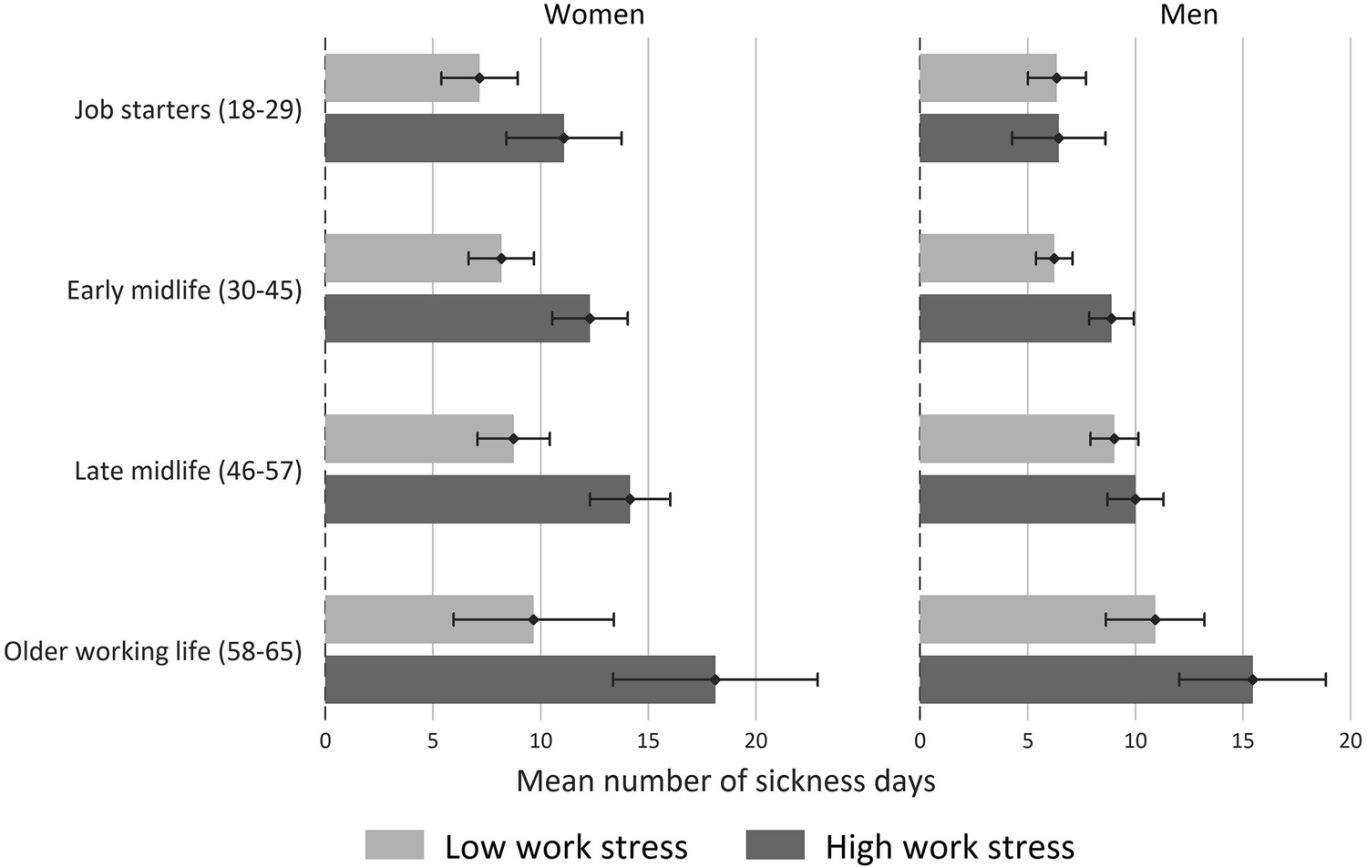
Predicted number of sickness days by work stress (ER ratio > 1) and age groups for women and men with 95% confidence intervals. Predicted scores are adjusted for partnership, years in current job, number of children living in the household, occupational position, education and income (Model 2 in Table 4)

At last, to summarize our main findings, we predict days of sickness absence based on multivariable regression models for each age group by levels of work stress (as exemplified by ER ratio > 1), and show predicted values in Fig. 1. All calculations and the graph are produced with Stata 14.

## Results

### Descriptive findings

The sample described in Tables 1 and 2 explores links between work stress and sickness days. Overall, the sample includes more men than women, and most workers belong to the two middle age groups (“early midlife” and “late midlife”). Respondents spent on average about 13 years in full-time education. Most respondents work in the lower service class or as manual worker (skilled or unskilled), live in a partnership, and are in good health. About 15% of the sample has an ER ratio above 1. The overall mean score of sickness days is 9.1 (with a standard deviation of 20.0). As we see in Table 2, medians of sickness days are generally smaller than the mean values, pointing to a right skewed distribution of sickness days.

### Distribution of work stress and sickness days by covariates

We see that work stress and sickness days vary by covariates under study: Men both have lower levels of work stress and fewer sickness days than women. In case of age, there is a positive association with sickness days, where number of days is higher in older age groups. Work stress, however, has an inverse u-shape association with age, with lower levels of work stress among youngest and oldest worker and higher values in the two middle age-categories (for each indicator of work stress). Turning to income and occupational position, it is worth noting that only low reward follows a social gradient (where working conditions are better for people with an advantaged social position). In case of sickness days, however, a disadvantaged occupational position is related to more sickness days. Finally, we see that days of sickness absence and levels of work stress are higher amongst people with poor health. In all cases, the reported associations are statistically significant, with *p* values below 0.05 (not reported in Table 2).

### Association between work stress and sickness days

Table 3 shows that sickness days are generally related to levels of work stress, not only in the total sample, but also within each age group. Yet, a closer look reveals three interesting findings: First, differences in sickness days appear somewhat larger for women than for men. Second, for women all indicators of work stress are clearly related to sickness days, while a relation between high effort and sickness days is not apparent for men. Third, it seems that differences are slightly more pronounced among older age groups (for men and women).

Table 4 presents results of the multivariable regressions based on non-transformed sickness days, and the results for transformed sickness days (square root) are presented in Table 5. For each of the indicators of work stress, three models with different adjustment sets were estimated. Since we present unstandardized coefficients (denoted as “b”), the estimates in Table 4 correspond to the adjusted mean differences in sickness days between those with and those without work stress. In sum, findings confirm the results from above and suggest that the reported associations are statistically significant (specifically for all age groups combined). Besides, four points are worth being noted: First, coefficients remain almost unchanged after accounting for social position in Model 2, thus, suggesting that links between work stress and sickness absence are not confounded by social position. Second, albeit coefficients remain statistically significant in most cases after inclusion of self-related health in model 3, estimates are generally attenuated. This suggests that parts of the association between work stress and sickness absence are due to poor health, but also that there is an independent effect. Third, when comparing estimates between the four age groups, they are somewhat higher in the oldest age group. Fourth, findings are consistent for non-transformed and transformed sickness days. Fig. 1 summarizes main findings, where days of sickness absence are predicted based on Model 2 in Table 4.

**Table 5:** Association between stress at work and sickness days

	Job starters (18–29)			Early midlife (30–45)			Late midlife (46–57)			Older working life (58–65)			All age groups		
	*b*	[95% CI]	*p*	*b*	[95% CI]	*p*	*b*	[95% CI]	*p*	*b*	[95% CI]	*p*	*b*	[95% CI]	*p*
Women															
High effort															
Model 1	0.20	[− 0.20, 0.59]	0.325	0.41	[0.14, 0.68]	0.003	0.56	[0.27, 0.86]	< 0.001	0.81	[0.12, 1.49]	0.021	0.44	[0.27, 0.62]	< 0.001
Model 2	0.18	[− 0.21, 0.58]	0.367	0.45	[0.18, 0.72]	0.001	0.62	[0.33, 0.92]	< 0.001	0.87	[0.17, 1.57]	0.014	0.48	[0.31, 0.66]	< 0.001
Model 3	0.05	[− 0.34, 0.44]	0.814	0.30	[0.04, 0.57]	0.027	0.39	[0.10, 0.69]	0.009	0.68	[0.02, 1.34]	0.042	0.31	[0.13, 0.48]	0.001
Low reward															
Model 1	0.46	[0.10, 0.82]	0.013	0.60	[0.35, 0.85]	< 0.001	0.72	[0.44, 1.00]	< 0.001	0.41	[− 0.28, 1.10]	0.240	0.61	[0.45, 0.78]	< 0.001
Model 2	0.44	[0.09, 0.80]	0.015	0.59	[0.34, 0.84]	< 0.001	0.70	[0.42, 0.98]	< 0.001	0.58	[− 0.11, 1.26]	0.101	0.60	[0.44, 0.76]	< 0.001
Model 3	0.31	[− 0.05, 0.67]	0.086	0.46	[0.21, 0.71]	< 0.001	0.50	[0.23, 0.71]	< 0.001	0.21	[− 0.45, 0.87]	0.532	0.43	[0.27, 0.59]	< 0.001
ER ratio > 1															
Model 1	0.11	[− 0.43, 0.64]	0.699	0.60	[0.26, 0.93]	< 0.001	0.82	[0.46, 1.17]	< 0.001	1.30	[0.43, 2.17]	0.003	0.66	[0.44, 0.87]	< 0.001
Model 2	0.07	[− 0.47, 0.61]	0.795	0.61	[0.28, 0.94]	< 0.001	0.84	[0.48, 1.19]	< 0.001	1.37	[0.50, 2.24]	0.002	0.67	[0.45, 0.88]	< 0.001
Model 3	− 0.17	[− 0.71, 0.36]	0.528	0.38	[0.05, 0.71]	0.023	0.57	[0.22, 0.92]	0.002	0.91	[0.07, 1.75]	0.033	0.42	[0.21, 0.64]	< 0.001
Men															
High effort															
Model 1	− 0.30	[− 0.68, 0.08]	0.126	0.02	[− 0.16, 0.19]	0.844	0.09	[− 0.12, 0.30]	0.387	0.31	[− 0.18, 0.79]	0.213	0.04	[− 0.08, 0.17]	0.491
Model 2	− 0.32	[− 0.70, 0.07]	0.106	0.08	[− 0.09, 0.26]	0.344	0.14	[− 0.07, 0.35]	0.184	0.44	[− 0.03, 0.92]	0.070	0.11	[− 0.02, 0.23]	0.094
Model 3	− 0.38	[− 0.77, 0.00]	0.051	− 0.01	[− 0.18, 0.16]	0.921	− 0.02	[− 0.23, 0.18]	0.825	0.29	[− 0.18, 0.75]	0.232	− 0.02	[− 0.14, 0.11]	0.811
Low reward															
Model 1	0.13	[− 0.23, 0.49]	0.471	0.53	[0.37, 0.69]	< 0.001	0.46	[0.26, 0.66]	< 0.001	0.50	[0.06, 0.94]	0.027	0.47	[0.35, 0.58]	< 0.001
Model 2	0.11	[− 0.24, 0.47]	0.536	0.48	[0.32, 0.64]	< 0.001	0.43	[0.23, 0.63]	< 0.001	0.41	[− 0.03, 0.84]	0.069	0.43	[0.31, 0.54]	< 0.001
Model 3	0.03	[− 0.33, 0.39]	0.861	0.36	[0.20, 0.52]	< 0.001	0.28	[0.09, 0.48]	0.005	0.19	[− 0.25, 0.62]	0.394	0.29	[0.18, 0.41]	< 0.001
ER ratio > 1															
Model 1	− 0.17	[− 0.76, 0.41]	0.565	0.56	[0.34, 0.78]	< 0.001	0.39	[0.13, 0.66]	0.004	0.83	[0.15, 1.50]	0.016	0.46	[0.30, 0.63]	< 0.001
Model 2	− 0.19	[− 0.77, 0.40]	0.529	0.54	[0.32, 0.76]	< 0.001	0.39	[0.13, 0.66]	0.004	0.84	[0.18, 1.50]	0.013	0.46	[0.30, 0.63]	< 0.001
Model 3	− 0.33	[− 0.93, 0.26]	0.270	0.40	[0.18, 0.62]	< 0.001	0.21	[− 0.05, 0.47]	0.115	0.62	[− 0.04, 1.27]	0.064	0.30	[0.14, 0.46]	< 0.001

Results of multivariable linear regression analyses predicting square rooted sickness days: regression coefficients (*b*), 95% confidence intervals [95% CI] and *p* values (*p*)

All estimations are based on random effect models accounting for year of data collection. Model 1 is adjusted for partnership–years in current job and number of children living in the household (and age in case of all age groups). Model 2 is additionally adjusted for occupational position, education and income. Model 3 additionally includes self-rated health

To formally test interactions between work stress and age groups, Table 6 (for non-transformed sickness days) and Table 7 (transformed sickness days) again investigates if work stress is linked to sickness absence across all ages (former Model 2), and then includes interactions between work stress and age groups (Model 2a). Two observations deserve attention: First, we again see that each measure of work stress is linked to increased number of sickness days, except of high effort in case of men. Second, once we include interactions in model 2 (indicating the difference in effects of work stress to the youngest age group), we observe that interactions are highest for the oldest age group, yet, they do not reach statistical significance. Thus, while effects tend to be higher for older workers, we cannot fully rule out that our findings of higher estimates in older groups are due to random errors. Again, findings are consistent for non-transformed and transformed sickness days.

**Table 6:** Interactions between stress at work and age group on sickness days

	High effort			Low reward			ER ratio > 1		
	*b*	[95% CI]	*p*	*b*	[95% CI]	*p*	*b*	[95% CI]	*p*
Women									
Model 2 [without interactions]									
High stress	4.65	[3.07–6.24]	< 0.001	4.85	[3.38–6.32]	< 0.001	6.40	[4.46–8.33]	< 0.001
Model 2a (with interactions)									
High stress (main effect)	3.14	[− 1.12–7.41]	0.149	2.98	[− 0.84–6.79]	0.126	3.12	[− 2.48–8.72]	0.275
High stress early midlife [30–45]	1.05	[− 3.89–5.99]	0.677	1.57	[− 2.89–6.02]	0.491	2.00	[− 4.37–8.38]	0.538
High stress late midlife [46–57]	1.60	[− 3.35–6.54]	0.526	2.71	[− 1.77–7.19]	0.235	4.10	[− 2.27–10.5]	0.207
High stress older working life [58–65]	5.61	[− 1.18–12.40]	0.105	2.82	[− 3.61–9.26]	0.390	10.2	[1.47–19.0]	0.022
*p* values of LR-test comparing model 1 and 2	0.403			0.658			0.097		
Men									
Model 2 (without interactions)									
High stress	0.86	[− 0.23–1.95]	0.123	2.46	[1.44–3.48]	< 0.001	3.33	[1.92–4.74]	< 0.001
Model 2a (with interactions)									
High stress (main effect)	− 1.05	[− 5.03–2.94]	0.607	− 0.72	[− 4.42–2.99]	0.704	− 1.32	[− 7.44–4.81]	0.673
High stress early midlife (30–45)	2.02	[− 2.28–6.32]	0.357	3.75	[− 0.25–7.75]	0.066	5.51	[− 0.95–12.0]	0.094
High stress late midlife (46–57)	1.52	[− 2.81–5.86]	0.491	2.71	[− 1.34–6.76]	0.189	3.42	[− 3.09–9.92]	0.304
High stress older working life (58–65)	4.29	[− 0.94–9.53]	0.108	4.75	[− 0.10–9.60]	0.055	8.74	[0.97–16.50]	0.028
*p* values of LR-test comparing model 1 and 2	0.391			0.196			0.074		

Results of multivariable linear regression analyses regression coefficients (*b*), 95% confidence intervals [95% CI] and *p* values (*p*)

Model 2 corresponds to Model 2 in Table 4 (all age groups). All estimations are based on random effect models accounting for year of data collection, and are adjusted for partnership, years in current job, number of children living in the household, occupational position, education and income

**Table 7:** Interactions between stress at work and age group on sickness days

	High effort			Low reward			ER ratio > 1		
	*b*	[95% CI]	*p*	*b*	[95% CI]	*p*	*b*	[95% CI]	*p*
Women									
Model 2 (without interactions)									
High stress	0.48	[0.31, 0.66]	< 0.001	0.60	[0.44, 0.76]	< 0.001	0.67	[0.45, 0.88]	< 0.001
Model 2a (with interactions)									
High stress (main effect)	0.22	[− 0.26, 0.69]	0.371	0.43	[0.01, 0.85]	0.045	0.09	[− 0.53, 0.71]	0.781
High stress early midlife (30–45)	0.21	[− 0.34, 0.75]	0.456	0.16	[− 0.33, 0.65]	0.528	0.48	[− 0.22, 1.19]	0.180
High stress late midlife (46–57)	0.34	[− 0.20, 0.89]	0.220	0.26	[− 0.24, 0.75]	0.311	0.72	[0.01, 1.43]	0.046
High stress older working life [58–65]	0.62	[− 0.13, 1.37]	0.108	0.092	[− 0.62, 0.80]	0.801	1.18	[0.21, 2.15]	0.017
*p* values of LR-test comparing model 1 and 2	0.387			0.770			0.081		
Men									
Model 2 (without interactions)									
High stress	0.11	[− 0.018, 0.23]	0.094	0.43	[0.31, 0.54]	< 0.001	0.46	[0.30, 0.63]	< 0.001
Model 2a (with interactions)									
High stress (main effect)	− 0.32	[0.77, 0.14]	0.175	0.10	[− 0.32, 0.53]	0.631	0.16	[0.86, 0.55]	0.662
High stress early midlife (30–45)	0.38	[− 0.11, 0.88]	0.126	0.37	[− 0.09, 0.82]	0.115	0.69	[− 0.05, 1.43]	0.069
High stress late midlife (46–57)	0.47	[− 0.03, 0.97]	0.064	0.30	[− 0.17, 0.76]	0.209	0.51	[− 0.23, 1.26]	0.178
High stress older working life (58–65)	0.72	[0.12, 1.32]	0.019	0.45	[− 0.10, 1.01]	0.110	1.15	[0.26, 2.05]	0.011
*p* values of LR-test comparing model 1 and 2	0.119			0.377			0.052		

Results of multivariable linear regression analyses predicting square rooted sickness days: regression coefficients (b), 95% confidence intervals [95% CI] and *p* values (*p*)

Model 2 corresponds to Model 2 in Table 5 (all age groups). All estimations are based on random effect models accounting for year of data collection, and are adjusted for partnership, years in current job, number of children living in the household, occupational position, education and income

## Discussion

This study used data from the GSOEP, collected among employed men and women in Germany, and investigated how stress at work (measured in terms of effort–reward imbalance and its two main components) is linked to subsequent number of sickness days (assessed 1 year later). In addition, the study compared associations of stress at work and sickness days between different age groups. According to these two research questions, two major findings result from our analyses: First, we found clear support that stressful work is linked to a higher number of sickness days. Yet, while this was true for each of the studied indicators in case of women (high effort, low reward and ER ratio > 1), we found no association for high effort in case of men. Importantly, associations persisted after accounting for three indicators of social position (education, income and occupational position), and additionally, they remained significant after adjusting for individual health at baseline. The second major finding was that associations were generally stronger amongst older worker, both for men and women. In analyses testing formally for effect modification, though, interactions between age and stress at work did not attained statistical significance.

Overall, our findings are in line with previous studies, specifically studies linking a effort–reward imbalance with sickness absence (Ala-Mursula et al. 2005; Derycke et al. 2013; du Prel et al. 2015; Fahlén et al. 2009; Lidwall 2016; Schreuder et al. 2010), but they also refine and add to existing knowledge in several ways:

First, by investigating associations among German full-time employees from various occupations and analysing sickness days in the year following the assessment of stress at work (1-year follow-up period), we extend existing evidence that was so far restricted to cross-sectional findings (du Prel et al. 2015) or to homogeneous occupational cohorts (Cunradi et al. 2005; Derycke et al. 2013; Donders et al. 2012; Farquharson et al. 2012; Head et al. 2007; Schreuder et al. 2010). Our results suggest that the associations between effort–reward imbalance and sickness days exist across a wide spectrum of jobs with varying incomes and educational levels. A next step would be to explore if the associations (albeit existing across different jobs) vary by occupational groups. To our knowledge, however, no such study exists so far [but only studies that investigate if social position moderates the association between work stress and health (Kuper et al. 2002; Rugulies et al. 2012)].

Second, we found that associations between work stress and sickness absence are slightly higher among older workers. This again extends existing knowledge, which at this point—to the best of our knowledge—is restricted to one cross-sectional study (Donders et al. 2012). However, in this study, the measure of work stress was not based on an explicit theoretical model and the sample was rather selective (employees of a Dutch university). The finding of a slightly stronger relationship for older people may have different reasons. Yet, it is premature to draw far-reaching conclusions about age per se. Rather, our study underlines that age is more than a chronological ageing process. In fact, it is a highly individualized process that incorporates changes of the physiological system and of socioemotional motivations (Carstensen et al. 1999), and resources (Hobfoll 1989), both with relevance for stress processing among older workers and the extent to which a person may feel a desire of being in control at work (Matschinger et al. 1986). Furthermore, the older people are, the more important it is to study stress processing in the light of previous live courses (Lazarus and DeLongis 1983). Specifically, coping skills may be less developed for people who have spent most of their life course in disadvantaged social and economic circumstances. For these people, chronic stress exposure could lead over time to deficient cognitive, emotional and social developments of core capabilities and coping skills (McEwen 2012), leaving them with higher vulnerability to chronic stress at work. Along these lines, future studies may not only study age differences of the associations between work stress and sickness days, but additionally consider factors from previous stages of the life course (Ben-Shlomo and Kuh 2002; Elder and Johnson 2002) as well as individual coping strategies and resources (Endler and Parker 1990; Scheibe and Zacher 2013).

Furthermore, because we found that high effort was not related to sickness absence for men (but for women), our study points to interesting sex differences. Perhaps, men who report a high level of effort feel very committed to their work [with a high “motivation to attend” (Steers and Rhodes 1978)], and therefore, they are less likely to be absent from work. Or, on a more conceptual level, this finding underlines that theoretical models of work stress should not focus on the level of psychological demands only, at least for men. Another reason, however, could simply be different response styles between men and women (i.e. social desirability) that trigger men to report a higher level of efforts at work than they actually have. Or, another explanation of why high effort does not lead to sickness absence in case of men, could be that men are more likely to recover from high efforts than women, because of traditional gender roles in the division of paid and unpaid work, leaving women with higher responsibilities beyond work (Casini et al. 2013; Laaksonen et al. 2008). At this point, an interesting question for future studies would again be if associations between work stress and sickness absence vary by occupational position, since coping skills, motivation and resources are probably more developed in advantaged positions.

Finally, because the association between work stress and sickness absence remained significant after accounting for individual health, our study raises the question what other pathways may underlay the observed association (besides health). Researchers, for example, have suggested that it is not only the low “ability to attend” that explains why stressful work leads to higher sickness days, but also that the “motivation to attend” matters as well (Steers and Rhodes 1978). In that case, for example, a stressful workplace may lead to a low motivation among employers, who in turn decide to avoid the stressful working environment (regardless of their health status).

Despite several strengths of our study (large study sample, theory-based assessment of stress at work, 1-year follow up), we have to consider several limitations. First, the measurement of work stress was restricted to one time point only. Because every person may be exposed to stress at some point in his or her life course, this not only includes people who are chronically stressed at work, but also people who are only occasionally stressed. As such, more comprehensive measures of work stress would be desirable, for example, repeated exposures or a measure that consider exposure duration as well. Second, our study was restricted to people who were full-time employed, and thus, we excluded an important fraction of todays’ labour market in Germany, that is, part-time employees, as well as employers and self-employed people. However, because self-employed people are not automatically eligible for statutory sick pay in Germany, they may represent a specific group of workers who deserve attention in another study. Third, some may argue that self-reported data on sickness absence (as used in our study) are prone to recall bias and less reliable than administrative records. Studies comparing both types of data, though, generally found high levels of agreement between both sources (Ferrie et al. 2005; Voss et al. 2008), as well as we maintain that self-reported data have many advantages compared with register data (e.g. they also include sickness absence that is not “officially” recorded). Fourth, we also must consider that sickness days were not normally distributed. Therefore, our multivariable analyses were additionally performed for transformed sickness days (square root) and we included non-parametric tests when studying bivariate associations. Also, three types of sensitivity analyses were additionally conducted (not shown): We replicated our main findings using both log-transformation of sickness days and negative binomial regressions. Furthermore, we performed all analyses with an alternative binary outcome measuring long-term sickness absence (30 days or more). Again, results were similar to the one reported and further supported our findings. As fifth limitation, we must consider that our analysis is based on data from Germany collected in 2006 and 2011, thus, we need to ask if results apply to today’s workforce or to other countries with different regulations for sickness benefits. Yet, at least for Germany, national regulations have not changed since 2006, including period with continued salary and benefit generosity. Sixth, the study did not consider the reason for sickness absence, for example, whether it was due to musculoskeletal disorders, accident, or to mental health problems. Indeed, we could ask if findings differ by reasons of sickness absence. Yet, GSOEP does not collect information on sickness absence reasons, and to investigate these questions in more detail, we clearly need larger sample sizes allowing meaningful analyses of subgroups. Finally, since the analyses excluded people who were not working at baseline, we may have excluded people who were in long-term sickness absence at baseline. It is, therefore, likely that we both underestimated the level of sickness absence and the association between work stress and sickness absence. Another limitation is that our analysis relies on one of the established work stress models only. Other measures of work stress, however, are not available in the data (Karasek et al. 1998).

In sum, this study shows that work stress, as measured in terms of effort–reward imbalance, is linked to higher number of sickness absence, and that these effects of work stress on sickness absence tend to be higher among older workers. One implication is that policies aiming at increasing the workability of older workers should aim at creating age-friendly workplaces, and pay particular attention to older workers. A second, rather conceptual implication is that future studies on age-differences need to recognize that age represents a complex category that involves numerous, often varying experiences from previous life courses.

### Electronic supplementary material

Below is the link to the electronic supplementary material.

Supplementary material 1 (DOCX 12 KB)

## Appendix: Sensitivity analyses

See Tables 8, 9 and 10.

**Table 8:** Association between stress at work and sickness days

	Job starters (18–29)			Early midlife (30–45)			Late midlife (46–57)			Older working life (58–65)			All age groups		
	*b*	[95% CI]	*p*	*b*	[95% CI]	*p*	*b*	[95% CI]	*p*	*b*	[95% CI]	*p*	*b*	[95% CI]	*p*
Women															
High effort															
Model 1	0.07	[− 0.17, 0.31]	0.581	0.20	[0.04, 0.36]	0.012	0.31	[0.14, 0.48]	< 0.001	0.34	[− 0.05, 0.72]	0.084	0.22	[0.12, 0.32]	< 0.001
Model 2	0.04	[− 0.21, 0.28]	0.765	0.22	[0.06, 0.37]	0.007	0.34	[0.17, 0.50]	< 0.001	0.40	[0.02, 0.79]	0.042	0.23	[0.13, 0.33]	< 0.001
Model 3	− 0.06	[− 0.31, 0.18]	0.617	0.13	[− 0.02, 0.29]	0.092	0.21	[0.05, 0.37]	0.013	0.30	[− 0.07, 0.66]	0.110	0.13	[0.035, 0.23]	0.008
Low reward															
Model 1	0.28	[0.06, 0.50]	0.011	0.35	[0.20, 0.49]	< 0.001	0.38	[0.22, 0.53]	< 0.001	0.16	[− 0.22, 0.54]	0.397	0.34	[0.24, 0.43]	< 0.001
Model 2	0.27	[0.05, 0.49]	0.016	0.34	[0.20, 0.49]	< 0.001	0.37	[0.22, 0.53]	< 0.001	0.25	[− 0.13, 0.64]	0.192	0.33	[0.24, 0.42]	< 0.001
Model 3	0.18	[− 0.04, 0.40]	0.100	0.27	[0.13, 0.41]	< 0.001	0.26	[0.11, 0.42]	0.001	0.051	[− 0.31, 0.41]	0.786	0.23	[0.14, 0.33]	< 0.001
ER ratio > 1															
Model 1	− 0.06	[− 0.38, 0.27]	0.738	0.31	[0.11, 0.50]	0.002	0.41	[0.21, 0.61]	< 0.001	0.54	[0.06, 1.02]	0.028	0.32	[0.19, 0.44]	< 0.001
Model 2	− 0.09	[− 0.42, 0.23]	0.571	0.31	[0.12, 0.50]	0.001	0.42	[0.22, 0.62]	< 0.001	0.64	[0.16, 1.13]	0.010	0.32	[0.20, 0.44]	< 0.001
Model 3	− 0.26	[− 0.58, 0.06]	0.117	0.18	[− 0.01, 0.37]	0.061	0.27	[0.07, 0.47]	0.007	0.39	[− 0.08, 0.85]	0.104	0.18	[0.06, 0.30]	0.003
Men															
High effort															
Model 1	− 0.22	[− 0.47, 0.02]	0.068	− 0.00	[− 0.11, 0.10]	0.934	0.08	[− 0.04, 0.20]	0.192	0.17	[− 0.09, 0.43]	0.187	0.03	[− 0.05, 0.10]	0.500
Model 2	− 0.24	[− 0.48, 0.01]	0.056	0.03	[− 0.07, 0.14]	0.546	0.10	[− 0.02, 0.23]	0.090	0.24	[− 0.01, 0.50]	0.064	0.06	[− 0.02, 0.13]	0.121
Model 3	− 0.28	[− 0.52, − 0.03]	0.026	− 0.02	[− 0.12, 0.08]	0.691	0.01	[− 0.11, 0.13]	0.889	0.16	[− 0.10, 0.41]	0.227	− 0.01	[− 0.08, 0.06]	0.755
Low reward															
Model 1	0.13	[− 0.10, 0.35]	0.265	0.32	[0.22, 0.41]	< 0.001	0.30	[0.18, 0.41]	< 0.001	0.28	[0.04, 0.52]	0.021	0.29	[0.22, 0.36]	< 0.001
Model 2	0.12	[− 0.10, 0.35]	0.279	0.29	[0.19, 0.39]	< 0.001	0.28	[0.17, 0.39]	< 0.001	0.23	[− 0.00, 0.47]	0.053	0.27	[0.20, 0.34]	< 0.001
Model 3	0.08	[− 0.15, 0.31]	0.505	0.23	[0.13, 0.32]	< 0.001	0.20	[0.09, 0.31]	0.001	0.11	[− 0.12, 0.35]	0.348	0.19	[0.13, 0.26]	< 0.001
ER ratio > 1															
Model 1	− 0.08	[− 0.45, 0.29]	0.661	0.31	[0.18, 0.44]	< 0.001	0.24	[0.09, 0.40]	0.002	0.48	[0.11, 0.84]	0.010	0.27	[0.18, 0.37]	< 0.001
Model 2	− 0.09	[− 0.46, 0.28]	0.636	0.30	[0.17, 0.44]	< 0.001	0.24	[0.09, 0.39]	0.002	0.47	[0.12, 0.83]	0.009	0.27	[0.18, 0.37]	< 0.001
Model 3	− 0.18	[− 0.55, 0.20]	0.355	0.22	[0.09, 0.35]	0.001	0.13	[− 0.02, 0.29]	0.079	0.35	[− 0.00, 0.70]	0.052	0.18	[0.09, 0.27]	< 0.001

Results of multivariable linear regression analyses predicting log transformed sickness absence [log (sickness absence + 1): regression coefficients (*b*), 95% confidence intervals [95% CI] and *p* values (*p*)]

All estimations are based on random effect models accounting for year of data collection. Model 1 is adjusted for partnership- years in current job and number of children living in the household (and age in case of all age groups). Model 2 is additionally adjusted for occupational position, education and income. Model 3 additionally includes self-rated health

**Table 9:** Association between stress at work and sickness days

	Job starters (18–29)			Early midlife (30–45)			Late midlife (46–57)			Older working life (58–65)			All age groups		
	IRR	[95% CI]	*p*	IRR	[95% CI]	*p*	IRR	[95% CI]	*p*	IRR	[95% CI]	*p*	IRR	[95% CI]	*p*
Women															
High effort															
Model 1	1.32	[0.98, 1.78]	0.068	1.20	[1.04, 1.38]	0.013	1.30	[1.13, 1.50]	< 0.001	1.80	[0.99, 3.25]	0.053	1.21	[1.11, 1.33]	< 0.001
Model 2	1.36	[1.00, 1.84]	0.050	1.20	[1.04, 1.38]	0.014	1.32	[1.14, 1.52]	< 0.001	1.62	[0.90, 2.91]	0.105	1.21	[1.11, 1.33]	< 0.001
Model 3	1.25	[0.92, 1.69]	0.161	1.13	[0.98, 1.30]	0.104	1.18	[1.02, 1.37]	0.025	1.39	[0.79, 2.46]	0.253	1.12	[1.02, 1.22]	0.018
Low reward															
Model 1	1.39	[1.06, 1.84]	0.018	1.36	[1.19, 1.56]	< 0.001	1.39	[1.21, 1.60]	< 0.001	0.79	[0.44, 1.44]	0.442	1.35	[1.24, 1.46]	< 0.001
Model 2	1.39	[1.05, 1.84]	0.021	1.36	[1.19, 1.55]	< 0.001	1.39	[1.21, 1.60]	< 0.001	1.09	[0.61, 1.97]	0.770	1.34	[1.23, 1.46]	< 0.001
Model 3	1.33	[1.01, 1.75]	0.045	1.28	[1.12, 1.46]	< 0.001	1.25	[1.08, 1.44]	0.002	0.76	[0.42, 1.36]	0.354	1.23	[1.13, 1.34]	< 0.001
ER ratio > 1															
Model 1	1.50	[0.98, 2.29]	0.059	1.27	[1.07, 1.50]	0.007	1.41	[1.20, 1.67]	< 0.001	1.82	[0.87, 3.78]	0.109	1.29	[1.15, 1.43]	< 0.001
Model 2	1.59	[1.04, 2.45]	0.033	1.26	[1.06, 1.49]	0.008	1.41	[1.19, 1.68]	< 0.001	1.85	[0.91, 3.74]	0.089	1.28	[1.15, 1.42]	< 0.001
Model 3	1.37	[0.86, 2.17]	0.186	1.15	[0.97, 1.36]	0.115	1.23	[1.04, 1.46]	0.018	1.30	[0.63, 2.68]	0.484	1.13	[1.01, 1.26]	0.028
Men															
High effort															
Model 1	0.79	[0.60, 1.03]	0.080	1.00	[0.90, 1.12]	0.963	1.10	[0.98, 1.23]	0.118	1.88	[1.24, 2.83]	0.003	1.04	[0.97, 1.11]	0.307
Model 2	0.78	[0.59, 1.01]	0.063	1.04	[0.93, 1.16]	0.523	1.11	[0.99, 1.25]	0.069	1.86	[1.24, 2.78]	0.003	1.06	[0.99, 1.14]	0.093
Model 3	0.74	[0.56, 0.97]	0.031	0.97	[0.87, 1.09]	0.645	1.01	[0.90, 1.13]	0.859	1.48	[0.99, 2.22]	0.058	0.99	[0.92, 1.06]	0.723
Low reward															
Model 1	1.15	[0.91, 1.45]	0.246	1.38	[1.25, 1.52]	< 0.001	1.31	[1.18, 1.46]	< 0.001	1.72	[1.22, 2.42]	0.002	1.33	[1.24, 1.42]	< 0.001
Model 2	1.16	[0.92, 1.47]	0.214	1.36	[1.23, 1.50]	< 0.001	1.31	[1.18, 1.46]	< 0.001	1.63	[1.17, 2.28]	0.004	1.31	[1.23, 1.40]	< 0.001
Model 3	1.11	[0.87, 1.42]	0.393	1.27	[1.15, 1.41]	< 0.001	1.21	[1.08, 1.35]	0.001	1.06	[0.75, 1.51]	0.738	1.22	[1.14, 1.30]	< 0.001
ER ratio > 1															
Model 1	0.95	[0.64, 1.40]	0.780	1.34	[1.17, 1.52]	< 0.001	1.25	[1.09, 1.44]	0.002	1.54	[1.16, 2.05]	0.003	1.30	[1.19, 1.42]	< 0.001
Model 2	0.92	[0.61, 1.37]	0.665	1.35	[1.19, 1.54]	< 0.001	1.26	[1.09, 1.45]	0.002	1.53	[1.15, 2.03]	0.003	1.31	[1.20, 1.43]	< 0.001
Model 3	0.82	[0.54, 1.24]	0.353	1.23	[1.08, 1.41]	0.002	1.13	[0.98, 1.31]	0.082	1.07	[0.64, 1.79]	0.808	1.18	[1.08, 1.29]	< 0.001

Results of multivariable negative-binominal regression analyses predicting number of sickness days: incidence rate ratios (IRR), 95% confidence intervals [95% CI] and *p* values (*p*)

All estimations are based on random effect models accounting for year of data collection. Model 1 is adjusted for partnership–years in current job and number of children living in the household (and age in case of all age groups). Model 2 is additionally adjusted for occupational position, education and income. Model 3 additionally includes self-rated health

**Table 10:** Association between stress at work and long-term sickness absence (> 29 days)

	Job starters (18–29)			Early midlife (30–45)			Late midlife (46–57)			Older working life (58–65)			All age groups		
	OR	[95% CI]	*p*	OR	[95% CI]	*p*	OR	[95% CI]	*p*	OR	[95% CI]	*p*	OR	[95% CI]	*p*
Women															
High effort															
Model 1	1.24	[0.53, 2.90]	0.622	2.25	[1.18, 4.30]	0.014	1.85	[1.12, 3.06]	0.016	2.93	[0.74, 11.6]	0.126	2.00	[1.43, 2.81]	< 0.001
Model 2	1.27	[0.56, 2.88]	0.561	2.92	[1.36, 6.26]	0.006	2.11	[1.26, 3.54]	0.005	4.03	[0.73, 22.2]	0.109	2.27	[1.60, 3.23]	< 0.001
Model 3	1.04	[0.45, 2.40]	0.931	2.20	[1.12, 4.30]	0.021	1.57	[0.97, 2.53]	0.066	3.40	[0.48, 24.2]	0.221	1.75	[1.24, 2.48]	0.001
Low reward															
Model 1	1.85	[0.79, 4.30]	0.155	2.23	[1.28, 3.88]	0.004	2.16	[1.33, 3.51]	0.002	2.37	[0.66, 8.56]	0.188	2.22	[1.61, 3.07]	< 0.001
Model 2	1.89	[0.92, 3.90]	0.084	2.28	[1.26, 4.14]	0.007	2.07	[1.26, 3.39]	0.004	3.09	[0.68, 14.1]	0.145	2.17	[1.56, 3.01]	< 0.001
Model 3	1.56	[0.74, 3.29]	0.242	1.73	[1.00, 3.00]	0.051	1.58	[0.99, 2.53]	0.053	2.07	[0.42, 10.4]	0.374	1.69	[1.22, 2.34]	0.002
ER ratio > 1															
Model 1	3.26	[0.30, 35.1]	0.329	2.33	[1.24, 4.40]	0.009	2.74	[1.53, 4.88]	0.001	5.26	[0.82, 33.7]	0.080	2.80	[1.92, 4.10]	< 0.001
Model 2	4.35	[0.48, 39.0]	0.190	2.65	[1.32, 5.33]	0.006	2.88	[1.60, 5.19]	< 0.001	7.83	[0.66, 92.3]	0.102	2.98	[2.02, 4.40]	< 0.001
Model 3	6.83	[0.36, 129.0]	0.200	1.89	[0.97, 3.70]	0.063	2.06	[1.20, 3.53]	0.008	4.75	[0.46, 49.3]	0.191	2.18	[1.48, 3.22]	< 0.001
Men															
High effort															
Model 1	0.95	[0.30, 2.96]	0.926	1.13	[0.76, 1.67]	0.555	1.13	[0.78, 1.62]	0.521	1.44	[0.87, 2.40]	0.156	1.17	[0.92, 1.49]	0.191
Model 2	0.96	[0.29, 3.14]	0.942	1.29	[0.86, 1.93]	0.214	1.23	[0.85, 1.78]	0.281	1.65	[0.98, 2.79]	0.061	1.32	[1.04, 1.68]	0.025
Model 3	0.88	[0.27, 2.87]	0.836	1.11	[0.74, 1.67]	0.622	1.00	[0.66, 1.49]	0.982	1.45	[0.85, 2.47]	0.174	1.11	[0.87, 1.42]	0.399
Low reward															
Model 1	0.51	[0.14, 1.78]	0.290	1.85	[1.28, 2.67]	0.001	1.78	[1.25, 2.52]	0.001	1.72	[1.07, 2.75]	0.025	1.76	[1.41, 2.21]	< 0.001
Model 2	0.43	[0.12, 1.57]	0.200	1.66	[1.15, 2.41]	0.007	1.70	[1.19, 2.43]	0.003	1.66	[1.03, 2.68]	0.038	1.64	[1.31, 2.05]	< 0.001
Model 3	0.39	[0.11, 1.40]	0.147	1.35	[0.93, 1.97]	0.116	1.49	[1.01, 2.19]	0.045	1.40	[0.85, 2.29]	0.182	1.37	[1.09, 1.72]	0.008
ER ratio > 1															
Model 1	0.69	[0.088, 5.38]	0.722	1.90	[1.23, 2.95]	0.004	1.56	[1.01, 2.43]	0.047	1.96	[1.04, 3.69]	0.038	1.75	[1.31, 2.32]	< 0.001
Model 2	0.65	[0.081, 5.26]	0.688	1.83	[1.18, 2.84]	0.007	1.59	[1.02, 2.48]	0.040	2.04	[1.07, 3.90]	0.030	1.75	[1.32, 2.32]	< 0.001
Model 3	0.57	[0.069, 4.66]	0.599	1.46	[0.93, 2.28]	0.100	1.31	[0.81, 2.10]	0.270	1.71	[0.88, 3.32]	0.111	1.41	[1.06, 1.88]	0.018

Results of multivariable logistic regression analyses predicting long-term sickness absence (30 days or more): odds ratios (OR), 95% confidence intervals [95% CI] and *p* values (*p*)

All estimations are based on random effect models accounting for year of data collection. Model 1 is adjusted for partnership years in current job and number of children living in the household (and age in case of all age groups). Model 2 is additionally adjusted for occupational position, education and income. Model 3 additionally includes self-rated health
