# Supplementary material for: Age differences in the association between stressful work and sickness absence among full-time employed workers: evidence from the German socio-economic panel
Source: Int Arch Occup Environ Health. 2018 Feb 28;91(4):479–96. doi: 10.1007/s00420-018-1298-3 (PMC5908813; doi:10.1007/s00420-018-1298-3)
Supplement: Supplementary file 1 — Supplementary material 1 (DOCX 12 KB) [file 420_2018_1298_MOESM1_ESM.docx]

**Supplementary Table**

Table S1. Items of the short-version of the Effort-reward Imbalance questionnaire

| **Dimension** | **Item** |
| --- | --- |
|  |  |
| Effort | - I have constant time pressure due to a heavy work load. |
|  | - I have many interruptions and disturbances while performing my job. |
|  | - Over the past few years, my job has become more and more demanding. |
| Reward | - I receive the respect I deserve from my superior or a respective relevant person. |
|  | - My job promotion prospects are poor |
|  | - I have experienced or I expect to experience an undesirable change in my work situation. |
|  | - My job security is poor. |
|  | - Considering all my efforts and achievements, I receive the respect and prestige I deserve at work. |
|  | - Considering all my efforts and achievements, my job promotion prospects are adequate. |
|  | - Considering all my efforts and achievements, my salary / income is adequate. |
|  |  |
